# Plasmodium ovale: Exploring an Atypical Presentation

**DOI:** 10.7759/cureus.74508

**Published:** 2024-11-26

**Authors:** Priscila Lopez, Samah Suleiman, Mario Valdez Imbert, Mark N Sayegh, Tjark Schliep

**Affiliations:** 1 Internal Medicine, Harlem Hospital Center, New York, USA; 2 Infectious Diseases, Harlem Hospital Center, New York, USA

**Keywords:** anopheles mosquito, malaria, parasitemia, plasmodium species, p ovale, shock

## Abstract

Malaria is an infection caused by five different Plasmodium species. The most common are *Plasmodium falciparum and vivax.* *Plasmodium ovale* is more rarely reported and mostly has a benign course. We present a case of a 40-year-old male with a six-day history of headaches, chills, and fever who was initially evaluated in our emergency room, from where he was discharged after a negative workup for malaria. He returned to the hospital five days later in septic shock. Now *P. ovale* was found in a blood smear. The patient was treated with three doses of intravenous artesunate, followed by a three-day course of oral atovaquone-proguanil. After G6P deficiency and sickle cell tests were found to be negative, he was started on primaquine for 14 days to eradicate liver hypnozoites. The patient had a favorable outcome; the pancytopenia resolved, and he remained stable and was discharged home.

## Introduction

Malaria is one of the most complex multisystem infections with a high morbidity and mortality. There are five different malaria species that infect humans: *Plasmodium falciparum*, *Plasmodium malariae*, *Plasmodium vivax*, *Plasmodium ovale*, and *Plasmodium knowlesi*. The infection is usually transmitted through the female Anopheles mosquito [1].

The malaria parasite life cycle involves two hosts. The Anopheles mosquito consumes infected blood from an infected host and then infects the next human with sporozoites. The sporozoites mature into schizonts in hepatocytes, where they can remain in a dormant state for months or even years in infections with *P. vivax* and *P. ovale*. Later, hepatocytes rupture and release dormant merozoites into the bloodstream and infect red blood cells; this cycle keeps repeating inside the red blood cells, where trophozoites turn to schizonts and get released as merozoites after red cell lysis into the bloodstream [1].

Cyclic fever, chills, nausea, vomiting, myalgias, headaches, and malaise are some of the symptoms associated with malaria infection that emerge in a cyclical manner every time merozoites are released into the bloodstream [1]. Malaria infection has been associated with various complications, including liver or renal impairment, cerebral malaria, and acute respiratory distress syndrome [2,3]. Around 200 million cases of malaria infection are reported annually, resulting in more than 600,000 deaths. More than 90% of these deaths occur in sub-Saharan Africa [4].

Based on our observations, there has been a recent increased incidence of malaria in New York City, coinciding with a large wave of immigration from Africa and South America.

## Case presentation

Our patient is a 40-year-old male with no significant past medical history who presented with complaints of headaches, subjective fever, chills, arthralgias, and malaise for six days. His symptoms were associated with an 8-10 pound weight loss over one month. He immigrated two months prior from Mauritania via Europe and then Central America, ultimately reaching the United States. He was treated for malaria infection two years prior in Mauritania.

A malaria rapid antigen screen (malaria protein antigen) and blood parasite smear were both negative in the emergency room. As the patient was hemodynamically stable with a temperature of 98.6 Fahrenheit, a heart rate of 82 beats per minute, a blood pressure of 110/72 mmHg, a respiratory rate of 19 breaths per minute, and an oxygen saturation of 98% on room air, he was discharged home with a diagnosis of viral-like syndrome, prescribed acetaminophen and ibuprofen, and instructed to return if symptoms worsen or fail to improve, as well as instructed to follow up with a primary care physician.

He presented to the emergency room for the second time five days later with worsening fever, malaise, headaches, and body aches. Upon presentation, his temperature was 101.3 degrees Fahrenheit, heart rate of 126 beats per minute, blood pressure of 113/53 mmHg, respiratory rate of 19 breaths per minute, and oxygen saturation of 97% on room air. The physical exam was normal with no nuchal rigidity, lymphadenopathy, organomegaly, or skin changes. The initial laboratory workup is shown in Table 1.

**Table 1:** Laboratory results HGB: hemoglobin; HCT: hematocrit; PLT: platelet; ALK PHOS: alkaline phosphatase; ALT (SGPT): alanine aminotransferase (serum glutamate pyruvate transaminase); AST (SGOT): aspartate aminotransferase (serum glutamate oxaloacetate transaminase); BUN: blood urea nitrogen; PCT: procalcitonin; PCR: polymerase chain reaction; CMIA: chemiluminescent microparticle immunoassay

Component	Reference range and units	Result
WBC	4.80-10.80 x 10(3)/mcL	2.79
RBC	4.70-6.10 x 10(6)/mcL	4.19
HGB	14.0-18.0 g/dL	12.2
HCT	42.0-52.0%	36.3
PLT	150-450 x 10(3)/mcL	55
Neutrophil %	44.0-70.0%	74.8
Sodium	136-145 mmol/L	134
Potassium	3.5-5.1 mmol/L	3.5
BUN	7-18 mg/dL	19
Creatinine	0.7-1.2 mg/dL	1
Magnesium	1.8-2.4 mg/dL	1.4
Albumin	3.97-4.94 g/dL	3.5
Total bilirubin	≤1.2 mg/dL	1.2
Direct bilirubin	0.0-0.3 mg/dL	0.4
ALK PHOS	40-129 U/L	54
ALT (SGPT)	≤41 U/L	23
AST (SGOT)	≤40 U/L	21
Lactate venous	0.6-1.4 mmol/L	1.2
C-reactive protein	0.0-5.0 mg/L	86.7
Procalcitonin (PCT)	0.02-0.08 ng/mL	8.66
HIV 1,2 AG/Ab by CMIA	Non-reactive	Non-reactive
SARS-CoV-2 PCR	Negative	Negative
Leptospira AB IgM by Dot Blot	Negative	Negative
Dengue IgM	<1.65 immune status ratio (ISR)	1.21

Malaria rapid screen negative (twice), but his blood parasite film (Giemsa stain) was now positive for *P. ovale *with 1% parasitemia. Blood cultures were negative. Chest X-ray was negative. In the emergency room, the patient became hypotensive with mean arterial pressures (MAPs) in the 50s requiring admission to the medical intensive care unit. He was managed for septic shock secondary to severe *P. ovale* malaria infection and was treated with lactated Ringer’s solution 2 liters, hydrocortisone 100 mg, and norepinephrine drip, as well as empiric broad-spectrum antibiotics. Intravenous artesunate 135 mg at hours 0, 12, and 24 hours, followed by oral atovaquone-proguanil 250 mg/100 mg for three days, was administered. After G6PD was ruled out, he was started on oral primaquine 0.25 mg/kg daily for 14 days for hypnozoite clearance. On day 8, he had fully recovered and was discharged home with a plan for outpatient follow-up.

## Discussion

*P. ovale*, among the five subtypes of malaria, has been shown to cause a mild and less severe form of the disease. Our case report is unique in that the aforementioned patient developed severe malaria secondary to the ovale subtype, necessitating the need for admission to the medical intensive care unit for vasopressor support despite adequate fluid resuscitation. Furthermore, our patient’s parasite density was low at 1%, making this case even more unusual.

*P. ovale* malaria is endemic to tropical Western Africa and has been less commonly reported in the Philippines, Indonesia, and Papua New Guinea [5,6]. In a systematic review from 2000 to 2020, data analysis from 113 studies showed that 51.33% were from the African region. The overall prevalence of *P. malariae* was 2.01%, which was higher than *P. ovale* spp. (0.77), which indicates that it has decreased in the last 20 years, but not significantly. No difference in prevalence between symptomatic and asymptomatic patients was observed for either *P. malariae *or *P. ovale* spp. [7]. *P. ovale* is the least common malaria species reported in the United States; the pooled prevalence from eight studies was 0.03 [8,9].

*P. ovale* is known to usually cause a mild disease with low levels of parasitemia. There are only a few cases of severe *P. ovale *infection published in the literature [10]. Jaundice, severe anemia, acute respiratory distress syndrome, and hypotension were the most common severe presentations described in *P. ovale* infections. A systematic review of severe *P. ovale* cases between 1922 and 2015 found that five cases out of 22 had a fatal outcome [9].

PCR is important in accurately differentiating Plasmodium species. Microscopy has a low sensitivity for detecting low-density infections, leading to missed diagnoses and the potential for misidentification of Plasmodium species [11].

Microscopic examination of thin and thick blood smears remains the diagnostic test of choice for *P. ovale* infections, as RDTs may not provide reliable results [12].

Rapid diagnostic test (RDT) sensitivity for *P. ovale* is as low as 22%. Low parasitemia is associated with false-negative results from RDTs regardless of the malaria species. Microscopic blood smear examination is therefore preferable for a definitive diagnosis of *P. ovale* [13]. The low parasitemia and poor sensitivity of RDT for *P. ovale* most likely contributed to the delay in diagnosis of our patient.

Chloroquine and/or an artemisinin combination therapy (ACT) are used to treat uncomplicated *P. ovale*. Primaquine is required to eradicate the hypnozoite liver stages of *P. ovale* and should be started after the fever has subsided and a normal glucose-6-phosphate dehydrogenase status has been confirmed [6]. Current WHO guidelines for severe malaria recommend that intravenous artesunate is the treatment of choice [10]. The patient presented in this case was treated with intravenous artesunate for a total of three doses, followed by a three-day course of atovaquone-proguanil, and after a negative result of G6PD, he was initiated on primaquine for 14 days.

## Conclusions

Malaria is a parasitic disease that is most commonly caused by *P. falciparum* and *P. vivax* and rarely *P. ovale*. The latter usually causes mild disease, but on rare occasions, it can lead to a severe presentation with high morbidity and mortality. Microscopic examination of thin and thick blood smears remains the diagnostic test of choice for *P. ovale* infections, as RDTs may not provide reliable results. In conclusion, early detection through proper testing allows for prompt diagnosis, preventing the progression of the disease and potential complications.
